# COVID-19 pandemic and other factors associated with unfavorable tuberculosis treatment outcomes—Almaty, Kazakhstan, 2018–2021

**DOI:** 10.3389/fpubh.2023.1247661

**Published:** 2023-09-21

**Authors:** Malika Gabdullina, Edmond F. Maes, Roberta Z. Horth, Panagul Dzhazybekova, Gulzhan N. Amanova, Sanam Zikriyarova, Dilyara A. Nabirova

**Affiliations:** ^1^Central Asia Field Epidemiology Training Program, Almaty, Kazakhstan; ^2^Department of Epidemiology, Asfendiyarov Kazakh National Medical University, Almaty, Kazakhstan; ^3^National Scientific Center of Phthisiopulmonology, Ministry of Health of the Republic of Kazakhstan, Almaty, Kazakhstan; ^4^Rollins School of Public Health, Emory University, Atlanta, GA, United States; ^5^United States Centers for Disease Control and Prevention, Central Asia Office, Almaty, Kazakhstan; ^6^Scientific and Practical Center for Sanitary and Epidemiological Expertise and Monitoring, Almaty, Kazakhstan

**Keywords:** COVID-19, drug-sensitive TB, tuberculosis treatment, unfavorable treatment, Kazakhstan

## Abstract

**Introduction:**

The COVID-19 pandemic negatively influenced the availability of tuberculosis (TB) services, such as detection, diagnosis and treatment, around the world, including Kazakhstan. We set out to estimate the COVID-19 pandemic influence on TB treatment outcomes by comparing outcomes among people starting treatment before the pandemic (2018–2019) and during the pandemic (2020–2021) and to determine risk factors associated with unfavorable outcomes.

**Methods:**

We conducted a retrospective cohort study among all people newly diagnosed with drug-sensitive pulmonary or extrapulmonary TB at least 18 years old who initiated treatment from 2018 to 2021 in Almaty. We abstracted data from the national electronic TB register. Unfavorable treatment outcomes were ineffective treatment, death, loss to follow-up, results not evaluated, and transferred. We used multivariable Poisson regression to calculate adjusted relative risk (aRR) and 95% confidence intervals (95%CI).

**Results:**

Among 1548 people newly diagnosed with TB during the study period, average age was 43 years (range 18–93) and 52% were male. The number of people initiating treatment was higher before than the pandemic (935 vs. 613, respectively). There was significantly different proportions before compared to during the pandemic for people diagnosed through routine screening (39% vs. 31%, *p* < 0.001), 60 years and older (16% vs. 22%, *p* = 0.005), and with diabetes (5% vs. 8%, *p* = 0.017). There was no difference in the proportion of HIV (8% in both periods). Unfavorable outcomes increased from 11 to 20% during the pandemic (aRR = 1.83; 95% CI: 1.44–2.31). Case fatality rose from 6 to 9% (*p* = 0.038). Risk factors for unfavorable TB treatment outcomes among all participants were being male (aRR = 1.44, 95%CI = 1.12–1.85), having HIV (aRR = 2.72, 95%CI = 1.99–3.72), having alcohol use disorder (aRR = 2.58, 95%CI = 1.83–3.62) and experiencing homelessness (aRR = 2.94, 95%CI = 1.80–4.80). Protective factors were being 18–39 years old (aRR = 0.33, 95%CI = 0.24–0.44) and 40–59 years old (aRR = 0.56, 95%CI = 0.41–0.75) compared to 60 years old and up.

**Conclusion:**

COVID-19 pandemic was associated with unfavorable treatment outcomes for people newly diagnosed with drug-sensitive TB in Almaty, Kazakhstan. People with fewer comorbidities were at increased risk. Results point to the need to maintain continuity of care for persons on TB treatment, especially those at higher risk for poor outcomes during periods of healthcare service disruption.

## Introduction

On March 11, 2020, the World Health Organization (WHO) declared Coronavirus Disease 2019 (COVID-19) to be a pandemic. In the immediate absence of an effective vaccine, “non-pharmaceutical interventions” (NPIs) such as social distancing, restrictions on travel, and remaining at home, were recommended as some of the main strategies to reduce the likelihood of disease transmission. With the exponential growth in the number of seriously ill people, these NPIs served as some of the main tools to reduce the immediate burden on the healthcare system personnel and resources (1). These restrictions and the demands placed on health care personnel (including personnel shortages) led to the postponement of elective health care procedures as well as decreased access to routine care, including the management of people with active tuberculosis (TB). Among countries with a large burden of TB, the reduction in core TB services led to reductions in the detection, diagnosis and treatment of patients with TB (2).

WHO estimates that in many countries with a heavy burden of TB, the number of TB notifications decreased by 18% in 2020 compared to 2019, as COVID-19 pandemic control measures were taken (2). The number of people under active treatment for TB globally also decreased in 2020, totaling 2.8 million people, 1.4 million fewer than in 2019.

**Figure 1:**
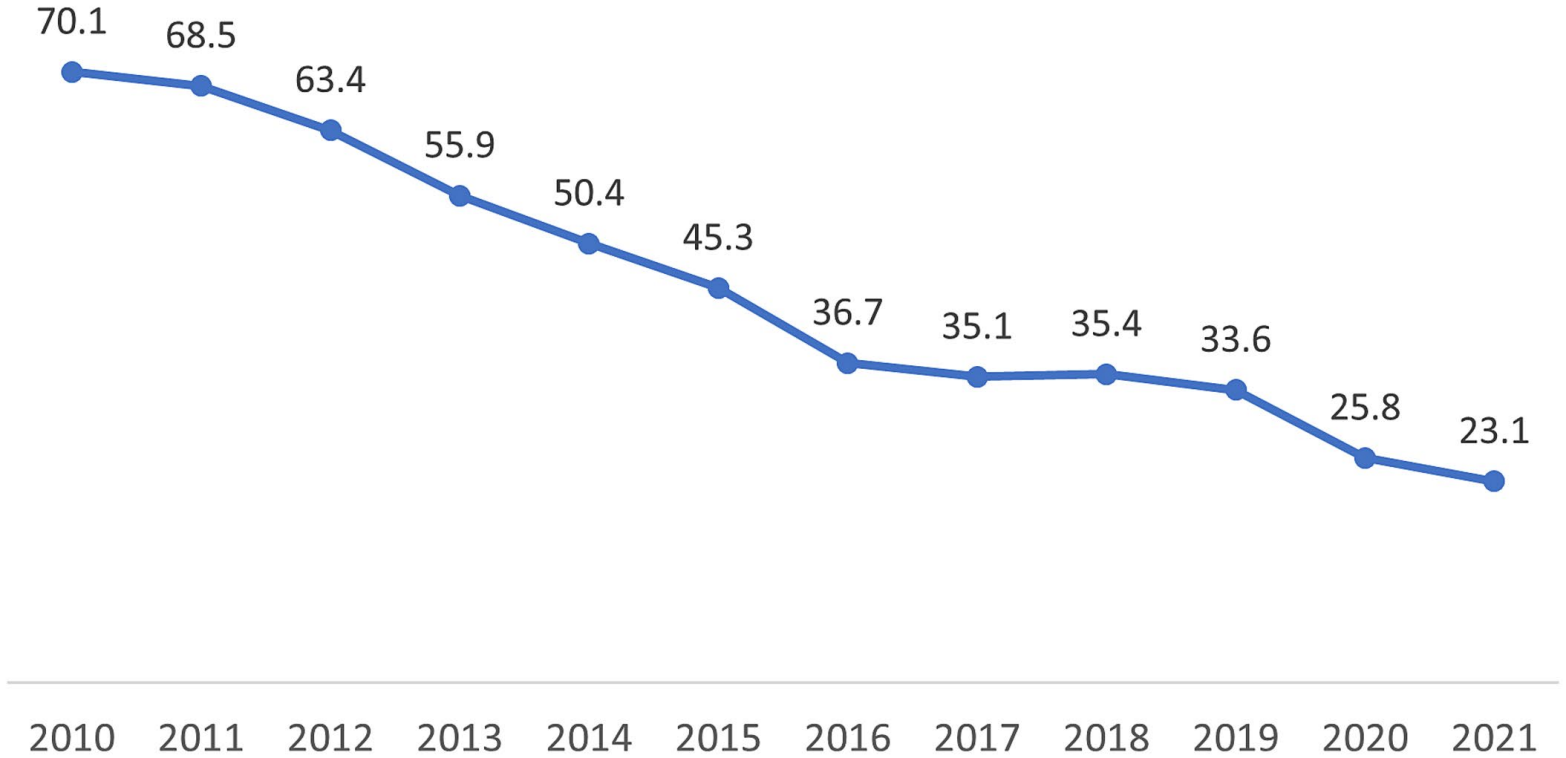
Annual incidence of tuberculosis per 100,000 population in Almaty, Kazakhstan, 2010–2021.

**Figure 2:**
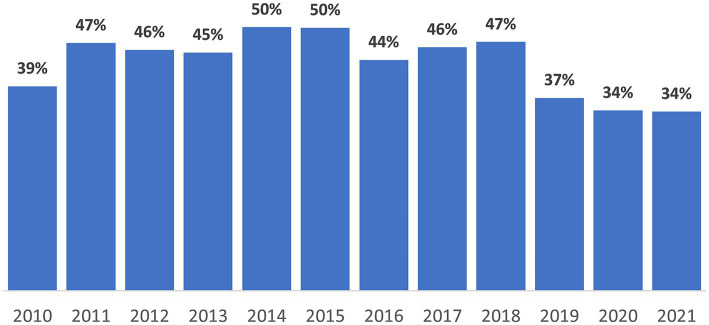
Annual proportion of new tuberculosis diagnosis from routine occupational screening in Almaty, Kazakhstan, 2010–2021.

After the introduction of the direct observed therapy strategy (DOTS) in 1999, the TB incidence per 100,000 people in Kazakhstan dropped from 162.5 in 2002 to 49.2 in 2020–an overall average decline of about 8–10% per year (3). Also, national TB mortality per 100,000 population decreased from 39.7 in 1999 to 1.9 in 2020. In Almaty, incidence decreased from 70.1 to 23.1 per 100,000 from 2010 to 2021 (Figure 1). From 2010 to 2019, the proportion of TB patients identified during occupational screening fluctuated between 38.8 to 36.6%; during 2020 and 2021, occupational screening only identified 34.2 and 34.0%, respectively (Figure 2).

The progress made in fighting TB in Kazakhstan, as well as worldwide, has been threatened by the COVID-19 pandemic. In particular, the pandemic has led to a decrease in the timely detection of TB in 2020 due to complex factors that resulted in reduced access to services (4). Specific impacts in Kazakhstan include: (1) reduced coverage of the population by preventive TB examinations (44.5% in 2020 compared to 41.9% in 2019), and (2) reduced detection of TB during routine medical check-ups (49.8 to 44.9 per 100,000 population in 2019 to 2020 respectively) (5).

A review of studies on the impact of the COVID-19 pandemic on TB services in various countries revealed that the COVID-19 pandemic negatively affected many aspects of TB control. In India, during the 8-week isolation due to the COVID-19 pandemic, the detection of TB decreased by 59% (6). In China, the diagnosis of multiple-drug-resistant (MDR) TB in the first quarter of 2020 decreased by 17% compared to the same period in 2019 (7). A study in Iran also showed a 55.6% decrease in new TB case detection during the March to June 2020 lockdown compared to previous years (8). A recent study in Italy showed that, despite efforts to maintain TB services, there was a sudden increase in service disruption during the COVID-19 outbreak (9). These service interruptions will likely have long-term consequences on TB burden, and a modeling study predicts a 4% increase in TB deaths worldwide and 5.7% excess deaths in India over the period from 2020 to 2025 due to the COVID-19 lockdown (10–12).

Studies using national data to assess the impact of the COVID-19 pandemic on TB services have not previously been conducted in any Central Asian country. This study examined the potential impact of COVID-19 on TB detection and treatment in Kazakhstan and will help guide recommendations for further planning and policy development of TB control programs in Kazakhstan, as well as other countries with similar economies and health care systems.

The specific aim of the study was to assess the association of the COVID-19 pandemic period and related risk factors with adverse TB treatment outcomes among people newly diagnosed with TB in Almaty, Kazakhstan, 2018–2021.

## Materials and methods

### Study design

We conducted a retrospective cohort study among people with newly diagnosed TB in Almaty; data were abstracted from patient registries between 08/20/2022 and 12/15/2022. Eligibility for this study was restricted to patients at least 18 years old, living in Almaty, with a first-time TB diagnosis who initiated TB treatment between 2018 and 2021.

### Data collection

Patient data was abstracted from Kazakhstan’s national electronic database - “Information System National Electronic Register of Tuberculosis Patients.” The database is a longitudinal registry where all people diagnosed with TB are mandatorily registered and tracked. The system contains demographic and clinical data on all people ever diagnosed with TB in Kazakhstan.

### Study participants

From 2018 to 2021 a total of 2,246 patients with TB were registered in Almaty, Kazakhstan. Analysis was restricted to 1,548 adults 18 years old and above, who were diagnosed for the first-time with drug-sensitive TB. People meeting these criteria without an individual identification number (*n* = 24) were excluded from the study.

To assess the effect of the COVID-19 pandemic on unfavorable TB treatment outcomes, we included only people who initiated treatment and would have already completed treatment before the study began. We excluded people with drug-resistant TB because currently in Kazakhstan the duration of treatment for this group requires several years.

The study population was divided into two groups: people initiating treatment during the COVID-19 pandemic from March 1, 2020, to December 31, 2021 (the “during COVID-19 period” group), and people initiating treatment before the COVID-19 pandemic from January 1, 2018, to February 29, 2020 (the “before COVID-19 period” group) in Almaty.

### Key definitions

We used WHO categories and reporting framework for TB, 2013 revision (updated Dec 2014; Jan 2020) to classify treatment outcomes as favorable or unfavorable (13). People were classified as having favorable treatment outcomes if they were considered to be cured or completed treatment. The definition of cured was someone who became smear or culture negative in the last month of treatment and on at least one previous occasion. People were classified as having unfavorable treatment outcomes if they had any of the following outcomes: treatment failure or switched 2nd line treatment, death from any cause, and loss to follow-up or not evaluated. Treatment failure was defined as having completed treatment but remaining smear or culture positive after treatment completion.

Drug-sensitive TB is TB caused by mycobacteria whose strains are sensitive to first-line anti-TB drugs (rifampicin, isoniazid). MDR TB is TB caused by mycobacteria whose strains are resistant to at least rifampicin and isoniazid.

### Statistical analysis

We assessed the accuracy and completeness of the data by constructing a line-by-line list of patients in a separate database and sorting them according to the variables under study. Statistically significant value of p was set to (*p* < 0.05). We analyzed the data and performed statistical calculations using R version 4.2.2 (R Foundation for Statistical Computing, Vienna, Austria).

We calculate crude risk ratios (cRRs) and used the chi-square test to measure the relationship between each individual risk factor, including time of treatment initiation, patient characteristics, with treatment outcome (successful versus unsuccessful). Power to detect difference in proportion from *p*1 = 0.11 to *p*2 = 0.20 from unequal samples (*n*1 = 935 and *n*2 = 613) was 0.99. We ran bivariable and multivariable Poisson regression to assess the contribution of treatment period and risk factors to unfavorable treatment outcomes. We checked for multicollinearity and interactions between explanatory variables. None were found. Results are presented as adjusted risk ratios (aRRs) and 95% confidence intervals.

### Ethical considerations

Ethical approval of the study was received from the local ethical commission of the NAO Kazakh National Medical University named after S.D. Asfendiyarov, Kazakhstan. This activity was reviewed by the CDC and was conducted consistently with applicable United States federal law and CDC policy.^1^

Permission to conduct the study was granted by the Local Internal Review Board of the Kazakh School of Public Health and the Internal Review Board at the US Centers for Disease Control and Prevention. Patients’ informed consent was deemed not necessary, because this is a retrospective analysis of program data.

## Results

We identified 1,548 people who had been newly diagnosed and initiated treatment with drug-sensitive TB from 2018 to 2021. Of these, 60% did so before the COVID-19 pandemic and 40% during the pandemic. Mean age was 43 years old and 50% were 18–39 years old (Table 1). Distribution across age groups differed significantly by period, and a greater proportion of people were 18–39 years old before the pandemic than during the pandemic (52% vs. 46%, respectively). Half (52%) were male and sex did not differ by period of detection. While 58% of all patients in the study were unemployed, the proportion of patients unemployed was similar in the pre-pandemic and pandemic time periods (58% versus 60%, respectively). More people were detected during routine screening before the pandemic (39%) than during the pandemic (31%). Also, more people were detected due to the presentation of symptoms during the pandemic (68%) compared to the pre-pandemic period (60%). People newly diagnosed with drug-sensitive TB were more likely to have diabetes during the pandemic than before (8% vs. 5%).

**Table 1:** Socio-demographic and epidemiological characteristics of adults newly diagnosed with drug-sensitive TB, grouped by years at first registration before and during COVID-19 pandemic, 2018–2021, Almaty, Kazakhstan (*n* = 1548).

Characteristics	Total *n* (%)	Before the pandemic^1^ *n* (%)	During the pandemic^1^ *n* (%)	*P*-value^2^
Overall	1548 (100)	935 (60)	613 (40)	
Age, years				
18–39	768 (50)	488 (52)	280 (46)	**0.005**
40–59	498 (32)	299 (32)	199 (33)	
60+	282 (18)	148 (16)	134 (22)	
Sex				
Men	810 (52)	502 (54)	308 (50)	0.202
Women	738 (48)	433 (46)	305 (50)	
Means of TB diagnosis				
Routine screening	554 (36)	366 (39)	188 (31)	**<0.001**
Test following symptoms	977 (63)	560 (60)	417 (68)	**0.001**
Post-mortem testing	17 (1)	9 (1)	8 (1)	0.702
Employment				
Unemployed	905 (58)	539 (58)	366 (60)	0.452
Manual laborer	215 (14)	135 (14)	80 (13)	0.486
Retired	231 (15)	126 (14)	105 (17)	0.057
Student	87 (6)	61 (7)	26 (4)	0.073
Office worker^3^	61 (4)	46 (5)	15 (2)	**0.021**
Healthcare worker	18 (1)	8 (1)	10 (2)	0.250
Experiencing homelessness	18 (1)	13 (1)	5 (1)	0.430
Risk factors for TB				
Contact with TB patient	18 (1)	10 (1)	8 (1)	0.857
Living with HIV	123 (8)	77 (8)	46 (8)	0.671
Alcohol use disorder	41 (3)	27 (3)	14 (2)	0.574
Drug use disorder	8 (0.5)	2 (0.2)	6 (1)	0.091
Incarceration <2 years	3 (0.2)	2 (0.2)	1 (0.2)	0.999
Diabetes	97 (6)	47 (5)	50 (8)	**0.017**
Pregnant at diagnosis	20 (1)	14 (2)	6 (1)	0.513
Postpartum <1 year	48 (3)	32 (3)	16 (3)	0.452

^1^Before the COVID-19 pandemic = January 1, 2018 to February 29, 2020. During the COVID-19 pandemic = March 1, 2020 to December 31, 2021. ^2^From Pearson’s Chi-square test with Yates correction. ^3^Office worker category captures management, business or financial operations, computer and math, architecture and engineering, sciences, education, sales and related, office and administrative support. Unknown or missing responses are excluded from analysis. Bolded numbers represent *p*-values < 0.05.

The proportion of people completing treatment was lower during than before the pandemic (58% vs. 51%, respectively; Table 2). Also, more people were transferred to second-line treatment during the pandemic than before (7% vs. 2%, respectively). The proportion who died from TB or other causes was also significantly higher during (9%) than before the pandemic (6%). There was no significant difference by period of treatment initiation for other outcomes.

**Table 2:** Treatment outcomes among adults newly diagnosed with drug-sensitive TB in Almaty before and during the COVID-19 pandemic, Kazakhstan 2018–2021.

Treatment outcomes	Overall	Before the pandemic^1^	During the pandemic^1^	*P*-value^2^
	*n* = 1548	*n* = 935	*n* = 613	
	*n* (%)	*n* (%)	*n* (%)	
Favorable^3^	1322 (85)	833 (89)	489 (80)	**<0.001**
Cured	464 (30)	287 (31)	177 (29)	0.479
Treatment completed	858 (55)	546 (58)	312 (51)	**0.004**
Unfavorable^3^	226 (15)	102 (11)	124 (20)	**<0.001**
Treatment failure	92 (6)	32 (11)	60 (10)	**<0.001**
2nd line treatment	57 (4)	15 (2)	42 (7)	**<0.001**
Ineffective treatment	35 (2)	17 (2)	18 (3)	0.203
Died	115 (7)	59 (6)	56 (9)	**0.038**
Died from TB	35 (2)	18 (2)	17 (3)	0.356
Died other causes	80 (5)	41 (4)	39 (6)	0.109
Lost to follow-up	16 (1)	10 (1)	6 (1)	0.933
Result not evaluated	3 (0.2)	1 (0.1)	2 (0.3)	0.712

^1^Before the COVID-19 pandemic = January 1, 2018 to February 29, 2020. During the COVID-19 pandemic = March 1, 2020 to December 31, 2021. ^2^From Pearson’s Chi-square test with Yates correction. ^3^From WHO categories and reporting framework for TB, 2013 revision (updated Dec 2014; Jan 2020). Bolded values are *p*-value < 0.05.

People who were newly diagnosed with drug-sensitive TB and initiated on treatment during the pandemic period were 1.85 times more likely [95% confidence interval (CI) = 1.46 to 2.36] to experience an unfavorable outcome compared to people who started treatment prior to the pandemic period (Table 3). People who were 18 to 39 years of age or 40 to 59 years of age were less likely to have an unfavorable outcome (cRR = 0.36 and 0.74, respectively) compared to people who were 60 years or older at time of treatment initiation. Males were more likely to have an unfavorable outcome compared to females (cRR = 1.66). People who were living with HIV or who had alcohol use disorder were more likely to have unfavorable treatment outcome, cRR = 2.49 and 2.99, respectively compared to people without those conditions.

**Table 3:** Risk factors associated with unfavorable treatment outcome among adults newly diagnosed with drug-sensitive TB, Almaty, 2018–2021.

Characteristics	Total *n* = 1548	Favorable outcome^2^ *n* = 1322	Unfavorable outcome^2^ *n* = 226	cRR [95% CI]	aRR [95% CI]
Period of diagnosis^1^					
Before the pandemic	935 (60)	833 (89)	102 (11)	Ref.	Ref.
During the pandemic	613 (40)	489 (80)	124 (20)	**1.85 [1.46, 2.36]**	**1.83 [1.44, 2.31]**
Age, years					
18–39	768 (50)	701 (91)	67 (9)	**0.36 [0.26, 0.49]**	**0.33 [0.24, 0.44]**
40–59	498 (32)	408 (82)	90 (18)	**0.74 [0.56, 0.98]**	**0.56 [0.41, 0.75]**
60+	282 (18)	213 (75.5)	69 (24.5)	Ref.	Ref.
Sex					
Male	810 (52)	664 (82)	146 (18)	**1.66 [1.29, 2.14]**	**1.44 [1.12, 1.85]**
Female	738 (48)	658 (89)	80 (11)	Ref.	Ref.
Employment (ref. not in category)					
Unemployed	905 (57)	768 (84)	137 (16)	1.09 [0.85, 1.40]	
Healthcare worker	18 (1)	15 (83)	3 (17)	1.14 [0.40, 3.40]	
Manual laborer	215 (14)	196 (91)	19 (9)	**0.57 [0.36, 0.89]**	
Office worker	61 (4)	59 (97)	2 (3)	**0.22 [0.05, 0.85]**	
Retired	231 (15)	180 (78)	51 (22)	**1.66 [1.26, 1.20]**	
Student	87 (6)	86 (99)	1 (1)	**0.08 [0.01, 0.53]**	
Experiencing homelessness					
Yes	18 (1)	10 (56)	8 (44)	**3.12 [1.83, 5.30]**	**2.94 [1.80, 4.80]**
No	1530 (99)	1312 (86)	218 (14)	Ref	Ref
Contact with TB patient					
Yes	18 (1)	15 (83)	3 (17)	1.14 [0.40, 3.24]	
No	1530 (99)	1307 (85)	223 (15)	Ref.	
HIV positive					
Yes	123 (8)	83 (68)	40 (33)	**2.49 [1.87, 3.33]**	**2.72 [1.99, 3.72]**
No	1425 (92)	1239 (87)	186 (13)	Ref.	Ref.
Alcohol dependency					
Yes	41 (3)	24 (59)	17 (41)	**2.99 [2.04, 4.39]**	**2.58 [1.83, 3.62]**
No	1507 (97)	1298 (86)	209 (14)	Ref.	Ref.
Drug dependency					
Yes	8 (0.5)	6 (75)	2 (25)	1.72 [0.51, 5.74]	
No	1540 (99.5)	1316 (85)	224 (15)	Ref.	
Incarceration ≤2 years					
Yes	3 (0.2)	2 (67)	1 (33)	2.29 [0.46, 11.39]	
No	1545 (99.8)	1320 (85)	225 (15)	Ref.	
Diabetes					
Yes	97 (6)	78 (80)	19 (20)	1.37 [0.90, 2.09]	
No	1451 (94)	1244 (86)	207 (14)	Ref.	
Pregnant at diagnosis					
Yes	20 (1)	17 (85)	3 (15)	1.03 [0.36, 2.94]	
No	1528 (99)	1305 (85)	223 (15)	Ref.	
Postpartum <1 year					
Yes	48 (3)	46 (2)	2 (4)	0.28 [0.07, 1.09]	
No	1500 (97)	1276 (85)	224 (15)	Ref.	

cRR, crude relative risk; aRR, adjusted relative risk. Statistically significant values from Z-test of coefficients from Poisson regression are bolded. ^1^Before the COVID-19 pandemic = January 1, 2018 to February 29, 2020. During the COVID-19 pandemic = March 1, 2020 to December 31, 2021. ^2^From WHO categories and reporting framework for TB, 2013 revision (updated Dec 2014; Jan 2020). Unknown or missing responses are excluded from analysis. Bolded values are those with *p*-values <0.05.

Employment status had five categories that were significantly related to treatment outcome. People who were manual laborers compared to all other categories were less likely to have unfavorable outcome (cRR = 0.58). People who were office workers compared to all other categories were less likely to have unfavorable outcome (cRR = 0.22). People who were students compared to all other categories were less likely to have unfavorable outcome (cRR = 0.08). People who were experiencing homelessness compared to all other categories were more likely to have unfavorable outcome (cRR = 2.94). People who were retired compared to all other categories were more likely to have unfavorable outcome (cRR = 1.46).

After simultaneously adjusting for all significant risk factors from the bivariate analysis, the association between treatment period and unfavorable outcome was aRR = 1.78 (95%CI = 1.41–2.26). The adjusted risk of adverse treatment outcome remained higher in males compared to females (aRR = 1.46, 95%CI = 1.12–1.9, *p* = 0.012; Table 3). Risk of unfavorable outcome remained increased for people living with HIV (aRR = 2.40, 95%CI = 1.74–3.30, *p* < 0.001), having alcohol use disorder (aRR = 2.40, 95%CI = 1.70–3.40, p < 0.001), people experiencing homelessness (aRR = 2.70, 95%CI = 1.65–4.43, *p* = 0.007).

Protective factors for adverse treatment outcomes of drug-sensitive TB were younger age 18–39 years (aRR = 0.35, 95%CI = 0.23–0.51, *p* < 0.001) and age 40–59 years (aRR = 0.57, 95%CI = 0.40–0.83, *p* = 0.003) versus 60 or more years of age.

## Discussion

Our study found that the COVID-19 pandemic period was associated with unfavorable treatment outcomes among adults newly diagnosed with drug-sensitive TB treatment in Almaty, Kazakhstan. This impact remained even after adjusting for several other risk factors including age, sex, HIV status, alcohol use disorder and employment status.

### TB detection during the COVID-19 pandemic

The overall number of people diagnosed with TB was substantially lower in the first two-years of the pandemic compared to the 2 years before the pandemic. This is consistent with the annual trends in Almaty where there has been a decreasing trend in TB incidence over the last decade, from 70.1 per 100,000 in 2010 to 35.1 in 2017 (the year before our study began). While community control measures, like use hand and respiratory hygiene practices, and social distancing, taken at the onset of the pandemic may have contributed to the reduced transmission of tuberculosis (14), it should be noted that health service delivery disruptions and reduced access to care may have led to fewer screening opportunities and fewer TB incident cases during the pandemic (15, 16). Nevertheless, reduced screening and healthcare service disruptions may also have contributed to the decrease.

The proportion of people newly detected with drug-sensitive TB during routine screening was significantly less during the pandemic than before (2). Systematic screening for TB is a central component of the global strategy to end TB (17). Screening helps detect TB disease early and reduces the risk of unfavorable treatment outcomes. Restrictive lockdowns introduced nationally in Kazakhstan at the onset of the pandemic made it harder for people to leave their houses to go receive preventive healthcare services, including TB screening for people at increased risk of developing TB disease. Also, even if people could leave, preventive services were often not available, because of disruptions in provision of primary care services throughout the country, including Almaty, during this time. People may also have been reluctant to obtain preventive services due to the risk of getting COVID-19 in healthcare facilities because rates of COVID-19 were high among healthcare providers (18).

Not surprisingly, the proportion of people detected with TB who tested because of TB symptoms was higher during the pandemic. Respiratory symptoms of COVID-19 can be similar to those of TB. During the initial phase of the pandemic and before testing was widely available, all people with respiratory symptoms consistent with COVID-19 in Kazakhstan were hospitalized. TB diagnostic tests would have been performed as a differential diagnosis of COVID-19. This is also consistent with our finding that the proportion of people diagnosed with TB increased in groups at higher risk for COVID-19, specifically older populations and people with diabetes. These are two commonly known risk factors for severe COVID-19 (19, 20).

### TB treatment outcomes during the COVID-19 pandemic

As expected, our study showed a decrease in the proportion of people completing TB treatment successfully during the pandemic. In Kazakhstan, as in other countries, some TB hospitals and care facilities were reappropriated to provide inpatient care for COVID-19 patients. Similarly, healthcare providers who usually treat people with TB were often reassigned to care for people with COVID-19 (4). Further amplifying this shortage of services, was the increased morbidity of COVID-19 among providers themselves (21). The reassignment of providers away from TB services could have resulted in reduced oversight and continuity of care for directly observed therapy (DOT) services (22).

Our results are consistent with other studies that show the negative impact the COVID-19 pandemic has had on TB treatment outcomes (7, 9, 23). Disruptions in treatment during the pandemic, may also have contributed to the increased proportion of people who failed to complete treatment or who were referred to second line treatment.

Disruptions in treatment may have also contributed to increased mortality, which was 50% higher during the pandemic (9% during vs. 6% before the pandemic). Notably, the proportion whose death was not attributed to TB was increased. There is no information on the cause of death in the database, but COVID-19 may have played a role because patients with active pulmonary TB who acquire COVID-19 have a two times greater risk of COVID-19 mortality (24).

### Treatment outcomes

Treatment success rate in our study of 85% was below the 90% target set by WHO, but it is consistent with the global treatment success rate of 86% for new and relapse cases (2). However, the success rate is higher than the success rate for the European region of 72%. Also, the case fatality ratio of 7% in our study is within the WHO target of 10% set for 2020, and in line with the 2025 target of 6.5%. Although case fatality ratios are below targets, there was a significant increase in all-cause mortality among TB patients during the pandemic. The majority of deaths were not attributable to TB. From the data we cannot determine if COVID-19 was a risk factor for the increased fatality rate; however, studies elsewhere have demonstrated that people with TB are at greater risk of dying from COVID-19 (25, 26).

Consistent with literature, men were more likely than women to have an unfavorable treatment outcome, as were people 60 years and older compared to young and middle-aged adults (27). Delayed-care seeking behavior and smoking status, which we did not measure in our study, are known to contribute to sex differences in TB outcomes. Also consistent with literature was the finding that people with health comorbidities and less social stability, such as alcohol use disorder, HIV, and experiencing homelessness, are more likely to have unfavorable treatment outcomes compared to people without these disadvantages (28).

### Study limitations

Due to the retrospective study design based on available data, we are limited to the information that is entered into the electronic database. There may also be errors in the entry of information into the database by employees of medical organizations, such as incorrect clinical and demographic data, and incomplete completion of medical records. Also, because data is collected by medical providers, our results are subject to self-report bias for certain variables with high stigma, such as drug and alcohol use. This bias likely results in underestimation of alcohol and drug use disorder in our study. Also, some variables had few responses and should therefore be interpreted with caution. Our study also did not assess any direct interactions between TB and COVID-19 because there was no information or inconsistently captured information about COVID-19 in the database. This information was incorporated into the database after the study period. Lastly, as an observational study limited to variables that could be found in medical records, we cannot control for all factors that could have contributed to differences in TB outcomes pre and during the COVID-19 pandemic.

### Study results in context

Decrease in proportion of people being newly diagnosed with TB from routine screening including occupational health screening, point to the need for maintenance of these essential services during periods of public health emergencies. Service continuity plans that support health care facilities to minimize disruption and ultimately increase the resilience of health services during public health emergencies are needed in preparation for future healthcare crisis (29).

Although there was a decrease in successful TB treatment outcomes during the pandemic, several strategies were adopted during this time that may have mitigated further negative impacts. One strategy included improved triage of patients at primary care and hospital entry. All patients presenting with cough, chest complaints or fever were immediately separated, given respirators, or surgical masks if respirators were not available, and were tested for COVID-19, TB, pneumonia, and acute respiratory viral infections.

Another strategy included the adoption of polymerase chain reaction (PCR) and enzyme-linked immunosorbent assay (ELISA) for rapid testing for differential diagnosis of different respiratory illnesses. During the beginning of the pandemic, Kazakhstan adopted a modified algorithm for rapid laboratory diagnosis of COVID-19 and TB. Rapid diagnosis using PCR-based methods made it possible to almost immediately diagnosis TB and initiate appropriate treatment.

Lastly, the country scaled up video observation therapy for TB. In video observed therapy, healthcare providers observe patients taking their anti-TB medications daily using live or recorded video. Studies elsewhere have found that adherence to treatment is higher among patients on video observed therapy than compared to in-person direct observed therapy (30). In 2018, Kazakhstan began to provide TB patients with smartphones to keep communication with their healthcare providers. Then in 2020, Kazakhstan launched a program to provide smartphones to all TB patients throughout the country (31). The use of video of the observed treatment (VOT) therapy in Kazakhstan allowed clinical staff to continue TB treatment in outpatient settings without interruption during the COVID-19 pandemic. The use of digital technologies during the COVID-19 pandemic also made it possible for providers to maintain communication with patients: conduct online consultation, speak with patients by phone, via telemedicine and mobile messaging.

## Conclusion

The COVID-19 pandemic was associated with unfavorable treatment outcomes for people newly diagnosed with drug-sensitive TB in Almaty, Kazakhstan. People with comorbidities (HIV or alcohol use disorder) and those experiencing homelessness were at increased risk of unfavorable outcomes. Detection through routine screening was reduced and the case fatality rate among people on TB treatment was increased during the pandemic. Results point to the need for maintaining routine TB screening and continuity of care for people on TB treatment, especially people at the highest risk of unfavorable outcomes, during times of healthcare service disruptions due to public health emergencies like COVID-19.

## Data availability statement

The data analyzed in this study is subject to the following licenses/restrictions: The dataset for the study is owned by the government of Kazakhstan. Official requests for the data can be made on request to the government of Kazakhstan. Requests to access these datasets should be directed to gabdullina.malika@gmail.com.

## Ethics statement

Ethical approval of the study was received from the local ethical commission of the NAO Kazakh National Medical University named after N.N. S.D. Asfendiyarov, Kazakhstan. This activity was reviewed by the CDC and was conducted consistently with applicable United States federal law and CDC policy. The studies were conducted in accordance with the local legislation and institutional requirements. The ethics committee/institutional review board waived the requirement of written informed consent for participation from the participants or the participants’ legal guardians/next of kin because information was pulled retrospectively from medical records for programmatic purposes.

## Author contributions

MG, RH, and DN contributed to the study design and data analysis. MG, GA, and DN contributed to the design of the study methods. MG and PD organized the data collection. MG, RH, EM, and DN contributed to the interpretation of the data and results of the study and supported with drafting the manuscript. All authors contributed to the article and approved the submitted version.

## Funding

Support for this project was provided by the United States Centers for Disease Control and Prevention, Central Asia Field Epidemiology Training Program (CDC Cooperative Agreement GH20-2108) in Almaty, Kazakhstan. The sponsor had no role in designing the study, collecting and analyzing the data, deciding whether to publish or prepare the manuscript.

## Conflict of interest

The authors declare that the research was conducted in the absence of any commercial or financial relationships that could be construed as a potential conflict of interest.

## Publisher’s note

All claims expressed in this article are solely those of the authors and do not necessarily represent those of their affiliated organizations, or those of the publisher, the editors and the reviewers. Any product that may be evaluated in this article, or claim that may be made by its manufacturer, is not guaranteed or endorsed by the publisher.

## Author disclaimer

The findings and conclusions in this report are those of the author(s) and do not necessarily represent the official position of the United States Centers for Disease Control and Prevention.

## Footnotes

^1^See e.g., 45 C.F.R. part 46, 21 C.F.R. part 56; 42 U.S.C. §241(d); 5 U.S.C. §552a; 44 U.S.C. §3501 et seq.
